# A genomic biomarker-based model for cancer risk stratification of non-dysplastic Barrett’s esophagus patients after extended follow up; results from Dutch surveillance cohorts.

**DOI:** 10.1371/journal.pone.0231419

**Published:** 2020-04-13

**Authors:** S. J. M. Hoefnagel, N. Mostafavi, M. R. Timmer, C. T. Lau, S. L. Meijer, K. K. Wang, K. K. Krishnadath

**Affiliations:** 1 Center for Experimental and Molecular Medicine, Amsterdam UMC, University of Amsterdam, Amsterdam, The Netherlands; 2 Department of Gastroenterology and Hepatology, Amsterdam UMC, Amsterdam, The Netherlands; 3 Department of Pathology, Amsterdam UMC, University of Amsterdam, Amsterdam, The Netherlands; 4 Division of Gastroenterology and Hepatology, Mayo Clinic, Rochester, Minnesota, United States of America; Peking University Cancer Hospital and Institute, CHINA

## Abstract

Barrett’s esophagus is the only known mucosal precursor for the highly malignant esophageal adenocarcinoma. Malignant degeneration of non-dysplastic Barrett’s esophagus occurs in < 0.6% per year in Dutch surveillance cohorts. Therefore, it has been proposed to increase the surveillance intervals from 3 to 5 years, potentially increasing development of advanced stage interval cancers. To prevent such cases robust biomarkers for more optimal stratification over longer follow up periods for non-dysplastic Barrett’s patients are required. In this multi-center study, aberrations for chromosomes 7, 17, and structural abnormalities for c-MYC, CDKN2A, TP53, Her-2/neu and 20q assessed by DNA fluorescence in situ hybridization on brush cytology specimens, were used to determine marker scores and to perform clonal diversity measurements, as described previously. In this study, these genetic biomarkers were combined with clinical variables and analyzed to obtain the most efficient cancer prediction model after an extended period of follow-up (median time of 7 years) by applying Cox regression modeling, bootstrapping and leave-one-out analyses. A total of 334 patients with Barrett’s esophagus without dysplasia from 6 community hospitals (n = 220) and one academic center (n = 114) were included. The annual progression rate to high grade dysplasia and/or esophageal adenocarcinoma was 1.3%, and to adenocarcinoma alone 0.85%. A prediction model including age, Barrett circumferential length, and a clonicity score over the genomic set including chromosomes 7, 17, 20q and c-MYC, resulted in an area under the curve of 0.88. The sensitivity and specificity of this model were 0.91 and 0.38. The positive and negative predictive values were 0.13 (95% CI 0.09 to 0.19) and 0.97 (95% CI 0.93 to 0.99). We propose the implementation of the model to identify non-dysplastic Barrett’s patients, who are required to remain in surveillance programs with 3-yearly surveillance intervals from those that can benefit from less frequent or no surveillance.

## Introduction

Barrett’s esophagus (BE) is a detectable precursor lesion that predisposes for the highly malignant esophageal adenocarcinoma (EAC). BE offers the possibility for endoscopic surveillance and for early diagnosis and treatment of dysplastic changes and EAC.

Depending on the maximum length of the Barrett’s segment following the Prague classification [1], the Dutch guideline recently has recommended 5 yearly endoscopic surveillance for the great majority of BE patients. These patients have no dysplasia and a maximum Barrett length of < 3cm [2]. Overall, this novel surveillance algorithm may result in better cost effectiveness but against the cost of an increased number of interval cancers with disastrous outcomes for the affected patients. The yearly risk for developing EAC of patients with non dysplastic Barrett’s esophagus (NDBE) in endoscopic surveillance series is between 0.3% and 0.6% for developing EAC, and between 0.9 and 1.0% for progression to either HGD or EAC [3]. Increasing surveillance intervals will likely increase the risk for patients not to adhere to their acid suppressive therapy. Since acid suppressive therapy decreases the risk on malignant progression, the progression rate of NDBE patients that are not included in surveillance programs and not treated by PPIs is likely to be higher [4]. Clearly, it is of high importance to develop biomarkers able to predict progression to dysplasia or cancer many years before progression will occur in the low risk Barrett’s patient cohorts. In the future, these biomarkers could also be critical for the implementation of population screening programs. These programs are of importance to tackle the problem of the majority of Barrett’s patients not being diagnosed, because of lack of symptoms and the consequence of 90% of EAC being detected at late stage. In the near future population screening programs will aim at identifying all BE patients in high risk populations. As these screening programs will identify thousands of newly diagnosed BE patients [5], the implementation of biomarkers to improve BE surveillance programs will be of high importance.

Thus, there is a high need for risk stratifying tools to safely optimize surveillance intervals for the low risk Barrett’s esophagus patients, and which can be implemented in future screening programs.

Despite promising predictive values of diverse genomic markers in previous studies and increasing availability of NGS methods such as whole genome sequencing for the detection of such markers, in today’s clinical practice the only predictive marker for cancer progression for BE patients is TP53. TP53 however is a relatively late marker mostly identifying patients that already have developed low or high-grade dysplasia [6]. Only few prospective studies on biomarkers for truly NDBE cohorts have been performed [7–13]. Because of the low progression rate of these patients, these studies are time consuming and therefore difficult to conduct [14, 15]. Based on a set of frequently occurring mutations, we earlier performed several biomarker studies on a prospective multi-center Dutch cohort of patients with NDBE [7, 8]. In one of these studies baseline genetic diversity was an important risk predictor [7]. In another analysis we found a set of frequently occurring genetic abnormalities including CDKN2A loss, c-MYC gain, and aneusomy to be predictive for progression to HGD and cancer [8].

The concept of diversity measurements has been proposed earlier to assess cancer progression risk in Barrett’s patients [16]. This concept is based on the assumption that a higher diversity in genomic clones leads to a higher chance of containing or producing a ‘well-adapted’ clone able to drive carcinogenesis [7].

In the current study, we analzyed genetic diversity as a comparison to conventional genetic abnormalities in a large prospectively followed cohort of NDBE patients after extended follow-up with a median follow up time of 86 months, compared to a median follow up of 43 months [7] and 45 months [8] in earlier studies on this cohort. At the end we combined both types of markers and clinical factors, enabling a direct comparison of the prognostic value of both types of molecular markers [7, 8], in order to build a model to predict cancer risk over prolonged periods of follow up. To this end, diverse Cox regression models were tested.

## Materials and methods

### Study population

This study was approved by the Medical Ethical Committee of the Academic Medical Center Amsterdam. All patients that were included provided written informed consent. 334 patients of a multi-center Dutch BE cohort that were previously included in the two biomarker studies with baseline biomarker analyses for single markers as well as diversity markers performed between 2002 and 2013 [7, 8] and who were under routine endoscopic surveillance and prospective follow up were included in this study. All patients were diagnosed with NDBE at inclusion. Patients who received endoscopic treatment or who had dysplasia in the past or who progressed within 6 months of follow up were excluded. Baseline was defined by the date that the cytology specimen for molecular analysis was taken. All patients underwent routine endoscopic surveillance following the ACG guidelines [17]. Progressors were defined as patients diagnosed with High Grade Dysplasia (HGD) or Esophageal Adenocarcinoma (EAC) by at least two pathologists including at least one expert GI pathologist during follow up.

### DNA FISH for the assessment of genomic markers

The molecular markers were assessed by DNA fluorescence in situ hybridization (FISH) on brush cytology specimens as published previously [7, 8]. Briefly, in every patient at least 50 Barrett cells (intestinal metaplastic cells) were investigated for chromosomal aberrations using locus-specific probes from two panels. We previously found that the variance of most statistics stabilized when at least 50 cells were scored [7].

Set 1 included markers for CDKN2A (p16), TP53, ERBB2 (HER-2), and centromeric probes for chromosome 17 (CEP17), and set 2 included centromeric probes for chromosomes 7 (CEP7) and 17 (Abbott Molecular) and markers for 20q and c-MYC. The results of each Barrett cell were individually scored and entered in a database. An “individual marker” score was defined for each marker, using the percentage of cells representing the individual genetic abnormalities in a patient as a continuous variable. In addition, aneusomy was defined as the mean of the percentage of cells with an abnormal CEP17 count in both sets and the percentage of cells with abnormal CEP7, and was used as a surrogate marker to assess DNA ploidy changes.

### Calculation of diversity scores

For every patient, the number of cells with a particular combination of the genomic abnormalities was defined. This data reflects clone-size abundance and was used for calculating diversity scores. Several diversity scores were calculated including the Shannon and Simpson (complement and inverse) indices and normalized clone score as described earlier [8]. The Shannon index is calculated as: $S=\sum_{i=1}^{R}Pi\;\text{ln}\left(Pi\right)$.

The Simpson index is calculated as: $S^{\prime }=\;\sum_{i\;=\;1}^{R}Pi^{2}$.

Where P*i* is the proportion of cells with clone *i* divided by the total number of cells, and R is the number of different clones. And the inverse of and the complement to the Simpson’s index are given by: Si=1/S′ and Sc=1-S′.

The normalized clone score was calculated by dividing the number of different clones observed in a sample by the total number of cells in the sample [8]. In contrast to our previous studies, diversity scores were based on single, two, three or four markers from both marker sets, to carefully investigate which particular markers should be used for diversity calculations and potentially increase cost-effectiveness when these markers will be implemented in clinical practice.

All data underlying results in this manuscript can be found in S4 Table.

### Finding of the optimal prediction model based on diversity scores

We performed univariate cox regression with different diversity scores from 1–4 markers. Diversity score were calculated over clones defined by 1–4 markers either from set 1 with the markers CDKN2A, TP53, ERBB2 and CEP17 or from set 2 with markers for CEP7, CEP17, 20q and c-MYC.

To determine which model had the best prediction accuracy, we used a bootstrap sampling technique with 1000 replications by setting a training and test set. For this purpose Akaike Information Criterion (AIC) and integrated area under the curve (iAUC) for diverse regression models were calculated.

We considered the models with the highest median Area Under the Curve value to be the optimal prediction models for progression to HGD/EAC in this cohort. Hazard ratio’s ratios, 95% confidence interval (CI) and p-values for the best performing models were calculated with univariate Cox regression analyses. A p-value of the Wald and the more stringent likelihood ratio test < = 0.05 was considered statistically significant. Analysis was performed using the Survauc package within R statistical software [18].

### Univariate models for analysis of the individual genomic markers and clinical variables

Individual markers and aneusomy were given as the percentages of cells with a particular chromosomal aberration by FISH analysis.

For every marker and each clinical variable, univariate Cox regression models were defined for calculation of hazard ratios with 95% confidence interval (CI). Variables were considered to be significantly predictive for progression to HGD/EAC, if the p-value of the Wald test and the likelihood ratio test was < = 0.05.

### Comparison of multivariate Cox regression models based on diversity, individual genomic marker scores and the clinical variables

Next, we evaluated multivariate Cox regression models, with different combinations of diversity and marker scores and using the clinical variables that were (borderline) significant in the univariate analyses. Based on the number of progressors (= 32 events), multivariate models were allowed to include 3 covariates to prevent overfitting of the models. We compared nested models with and without splines for every covariate (splines were first applied to age, then to average Barrett circumferential length and then to the third covariate), and performed Anova testing (Chi-square). In case we found significant differences between the models, we used the model with the highest log likelihood ratio’s. For each model, we again used bootstrapping with 1000 replications for calculation of prediction accuracy with the Akaike Information Criterion and integrated Area Under the Curve [18].

The best performing model was the model with the highest integrated Area Under the Curve combined with the most acceptable Akaike Information Criterion, to make sure there was no overfitting.

### Leave-one-out analysis

To determine test accuracy of this best performing model, we performed a leave-one-out cross validation analysis on the entire cohort (n = 334). We trained the Cox prediction model on all patients except for 1, and calculated a risk score for the 1 patient left out of the training set. We divided the complete cohort based on the risk scores at different binary cut-offs. Sensitivity and specificity were calculated, and three cut-off values with increasing sensitivity were selected. Kaplan Meier analysis and log rank test were performed to compare the high and low risk groups defined by these three binary cut-offs of risk-scores defined by the model. The positive and negative predictive values and likelihood ratios of the models using the three cut-offs were calculated.

## Results

### Patient characteristics and progressors

Of the 428 patients from our earlier analyzed cohort [7], 3 patients were excluded because of a diagnosis of Low Grade Dysplasia at the date the brush specimen was taken and 91 patients were excluded because of unavailability of per cell FISH data or too low number of Barrett’s cells that were analyzed by FISH.

334 patients could be enrolled for the analyses because of the availability of DNA per single cell FISH scores, which is required for calculating diversity measures. All patients were diagnosed with NDBE at inclusion. The majority of patients were male (81%) and the median age was 60.0 years (interquartile range (IQR) = 15.75) at baseline endoscopy (Table 1). The median C-Barrett length was 2 cm and the median maximum length (M-Barrett length) was 4 cm, both measured at baseline. The majority of patients (53%) were long segment BE patients (>3 cm). The median prospective follow-up time of these patients was 86.5 months (IQR 39.7). 95% of the patients had a follow-up time longer than 3 years. A total of 32 (9.6%) of patients progressed to HGD or EAC (16 to HGD, 16 to EAC) after a median follow up time of 39.9 months (IQR 55.3).

The annual progression rate to HGD and/or EAC of the whole cohort was 1.3%, while progression to EAC was 0.85% per year.

**Table 1:** Cohort characteristics.

Characteristics	Entire cohort (n = 334)
Community hospitals (%)	65.9% (*n* = 220)
Male sex fraction	80.8% (*n* = 270)
Median age (IQR)	60.0 (15.75)
Median *Circumferential segment length (*C-Barrett length*)* in cm (IQR)	2 (4)
*longest tongue length > 3 cm* (Long Segment Barrett’s esophagus)	53% (n = 176)
Median *longest tongue length* (M-Barrett length) in cm (IQR)	4 (4)
Average BMI (± SD)	27.2±3.8
Use of proton-pump inhibitors	99.4% (*n* = 332)
Family history of Barrett’s esophagus versus no family history	11.3% (*n* = 37)
Family history of esophageal cancer versus no family history	8.3% (*n* = 27)
Smoking (current and former versus no smoking)	70.6% (*n* = 233)
Progressors	9.6% (*n* = 32)
Median time untill progression months (IQR)	39.9 (55.3)
Median time of follow-up of non progressors months (IQR)	90.2 (39.1)
Median time of follow-up complete cohort in months (IQR)	86.5 (39.7)

IQR, interquartile Range; LSBE, long segment Barrett’s esophagus; BMI, Body Mass Index; SD, standard deviation.

### Prediction of progression by measures of clonal diversity scores

Cox regression models with clonal diversity scores derived from calculations from 1–4 markers were compared, and the best performing models based on the highest integrated Area Under the Curve for prediction of HGD/EAC development are shown in S1 and S2 Tables. Median iAUC and median AIC were comparable between the models using 1–4 markers. The most significant diversity score from set 1 (markers CDKN2A/TP53/ERBB2/CEP17) was the normalized clone score (NC) over TP53/ERBB2 and CEP17. This was the only significant predictor in univariate analysis according to the Wald test (Table 2, HR 821491, Wald test p = 0.03). The normalized clone score over CEP7/ CEP17/20q/c-MYC was the best predictive diversity score derived from set 2, which showed a trend (Table 3, HR 66.79, Wald test p = 0.1). Both, the NC of TP53/ERBB2/CEP17 and the NC over the markers CEP7/CEP17/20q/c-MYC, were entered in multivariate regression analyses.

**Table 2:** Outcomes of the best performing clonal diversity score over set 1^†^.

Model	Median iAUC^1000 rep	Median AIC^1000 rep	P value Wald*	P value likelihood ratio *	HR*	95% CI*
**3 markers:** **NC TP53/ERBB2/CEP17**	**0.56**	**147**	**0.03**	**0.07**	**821491**	**3.44–1.96e+11**

^†^markers CDKN2A/TP53/ERBB2/ CEP17.

^ Obtained with bootstrapping.

* Results from univariate Cox proportional hazards models. Bold values indicate statistical significance (P<0.05).

iAUC, integrated Area Under the Curve; AIC, Akaike’s Information Criterion; HR, Hazard Ratio; CI, Confidence Interval; NC, Normalized Clone score.

**Table 3:** Outcomes of the best performing clonal diversity score over set 2^†^.

Model	Median iAUC^1000 rep	Median AIC^1000 rep	P value Wald*	P value likelihood ratio *	HR*	95% CI*
4 markers: NC CEP7/CEP17/ 20q/c-MYC	0.61	150	0.1	0.2	66.79	0.30–15024

^†^markers CEP7/CEP17/20q/c-MYC.

^ Obtained with bootstrapping.

* Results from univariate Cox proportional hazards models.

AUC, integrated Area Under the Curve; AIC, Akaike’s Information Criterion; HR, Hazard Ratio; CI, Confidence Interval; NC, Normalized Clone score.

### Univariate regression analysis of the clinical variables

Next, we performed univariate Cox regression analysis to examine the prognostic significance of the clinical parameters including age, BMI, C-Barrett length and M-Barrett length of the Barrett’s segment at baseline with respect to progression to HGD/EAC. For all clinical parameters, the continuous variables lead to a higher integrated Area Under the Curves than binary variables. Age was a significant predictor (Wald test p = 0.00049, likelihood ratio test p = 0.0002652), with a hazard ratio of 1.1 per gained year (Table 4). C-Barrett length did not achieve significance, but showed a trend (Wald test p = 0.059, likelihood ratio test p = 0.076), with a hazard ratio of 1.1 per gained cm Barrett’s segment. Age and C-Barrett length of the Barrett’s segment were used in the multivariate regression model.

**Table 4:** Outcomes from univariate regression analysis of the clinical variables.

Variable	Unit	P value wald*	P value likelihood ratio*	HR*	95% CI*
**Age**	**per year**	**0.00049**	**0.0002652**	**1.1**	**(1–1.1)**
**C-Barrett length**	**per cm**	**0.059**	**0.076**	**1.1**	**(1–1.2)**
M-Barrett’s length	per cm	0.079	0.097	1.1	(0.99–1.2)
BMI	per kg per m^2^	0.95	0.945	1	(0.91–1.1)

* Results from univariate Cox proportional hazards models. Bold values indicate statistical significance (P<0.05) and borderline results.

HR, Hazard Ratio; CI, Confidence Interval; BMI, Body Mass Index.

### Univariate regression analysis of the individual genomic markers and aneusomy

The covariates defined by the individual genomic markers scores were analysed in univariate Cox regression analysis to investigate their predictive value. The results are shown in Table 5. CEP7 gain was a significant predictor of progression to HGD/EAC according to the Wald test (p = 0.02), with an HR of 1.2 per 1% gain. CEP7 gain was used in the multivariate regression model.

**Table 5:** Outcomes from univariate regression analysis of the genomic markers.

Variable	Unit	Variable type	P value Wald*	P value likelihood ratio *	HR*	95% CI*
**CEP7 gain**	%	continuous	0.02	0.0179	1.22	**(1.03–1.43)**
c-MYC gain	%	continuous	0.2	0.206	1.12	(0.94–1.33)
CEP17 gain	%	continuous	0.4	0.395	1.05	(0.94–1.16)
CEP17 loss	%	continuous	0.5	0.466	0.91	(0.70–1.18)
20q gain	%	continuous	0.5	0.469	1.08	(0.87–1.34)
CEP7 loss	%	continuous	0.5	0.475	1.06	(0.91–1.23)
Aneusomy	%	continuous	0.5	0.497	1.02	(0.96–1.09)
ERBB2 gain	%	continuous	0.7	0.729	1.02	(0.91–1.15)
CDKN2A loss	%	continuous	0.7	0.689	0.99	(0.94–1.04)
TP53 loss	%	continuous	0.9	0.941	1	(0.88–1.3)

* Results from univariate Cox proportional hazards models. Bold values indicate statistical significance (P<0.05).

HR, Hazard Ratio; CI, Confidence Interval.

### Multivariate regression analyses using diversity scores, individual covariates and clinical parameters

**Fig 1:**
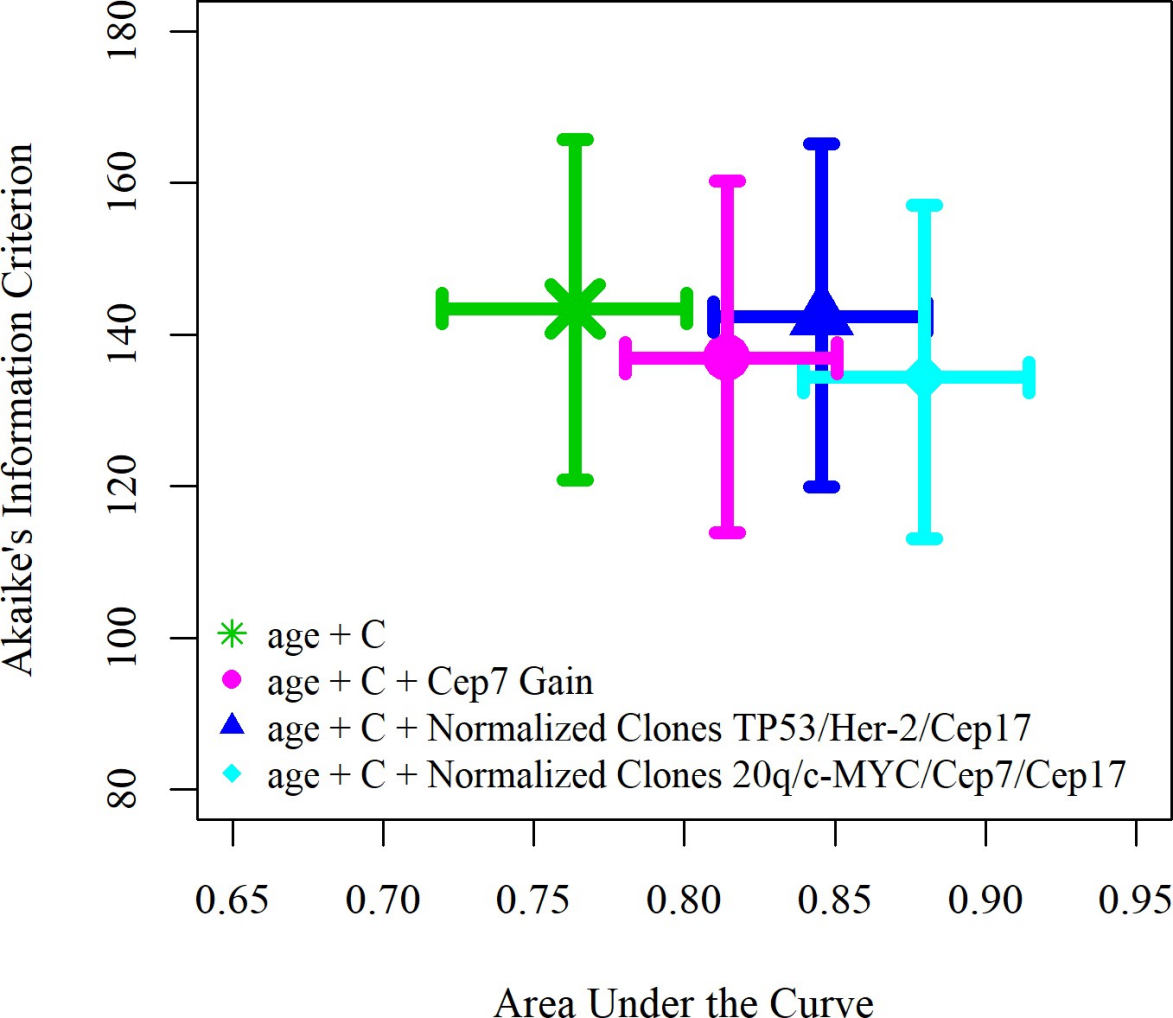
Area under the curve and Akaike Information Criterion of several models with a subset of variables in each model split up in multiple segments by splines. C, C-Barrett length.

A series of models, using splines for those variates that performed better in the model, were compared using multivariate regression analyses (Fig 1). In these models we included different combinations of the clinical, diversity and individual covariates that were found to be significant in the univariate analyses. Given the number of events, a maximum of 3 variables were included in each model to prevent overfitting.

**Fig 2:**
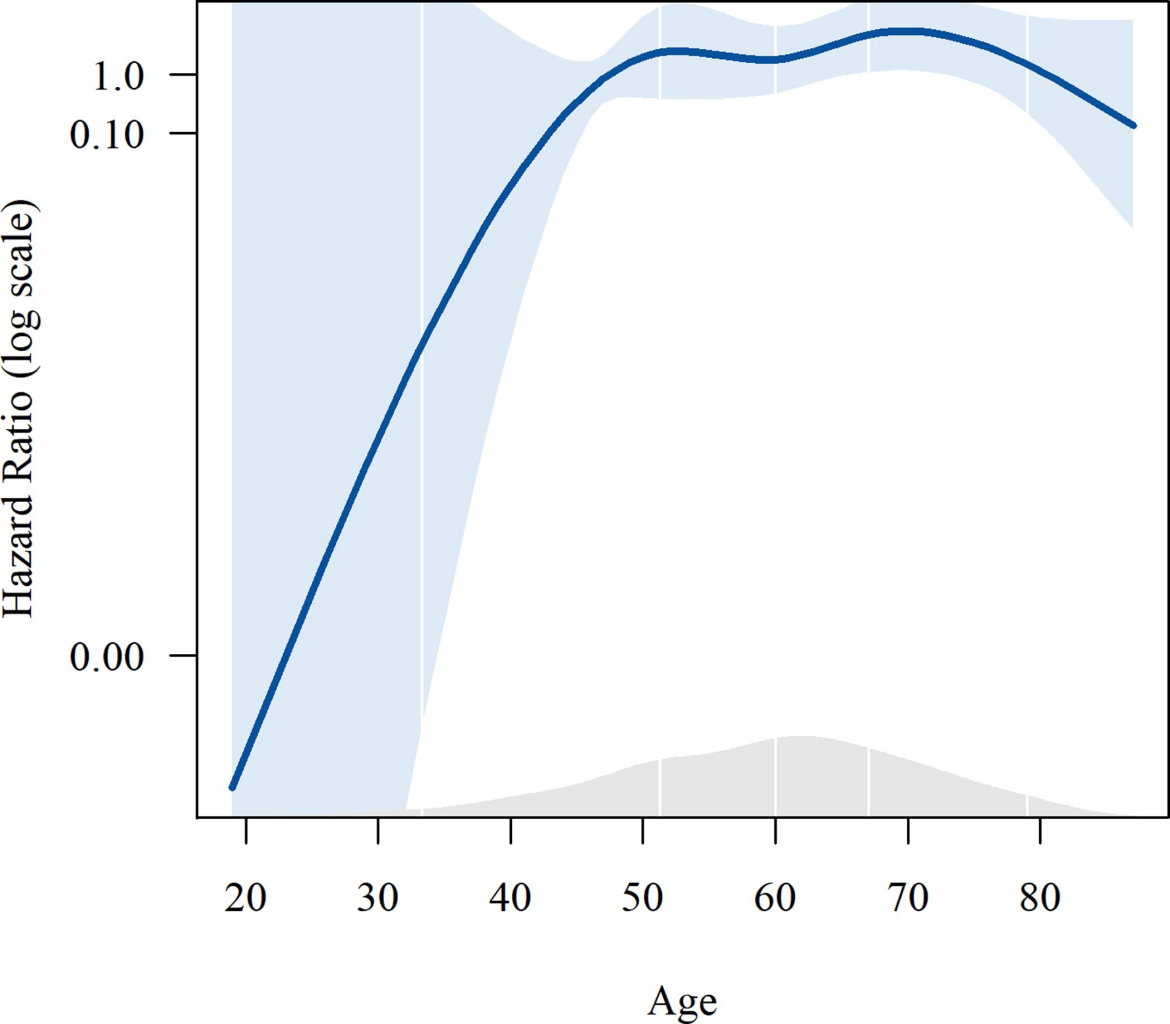
Plots of splines for age in the best performing multivariate model predicting progression in leave-one-out analysis.

**Fig 3:**
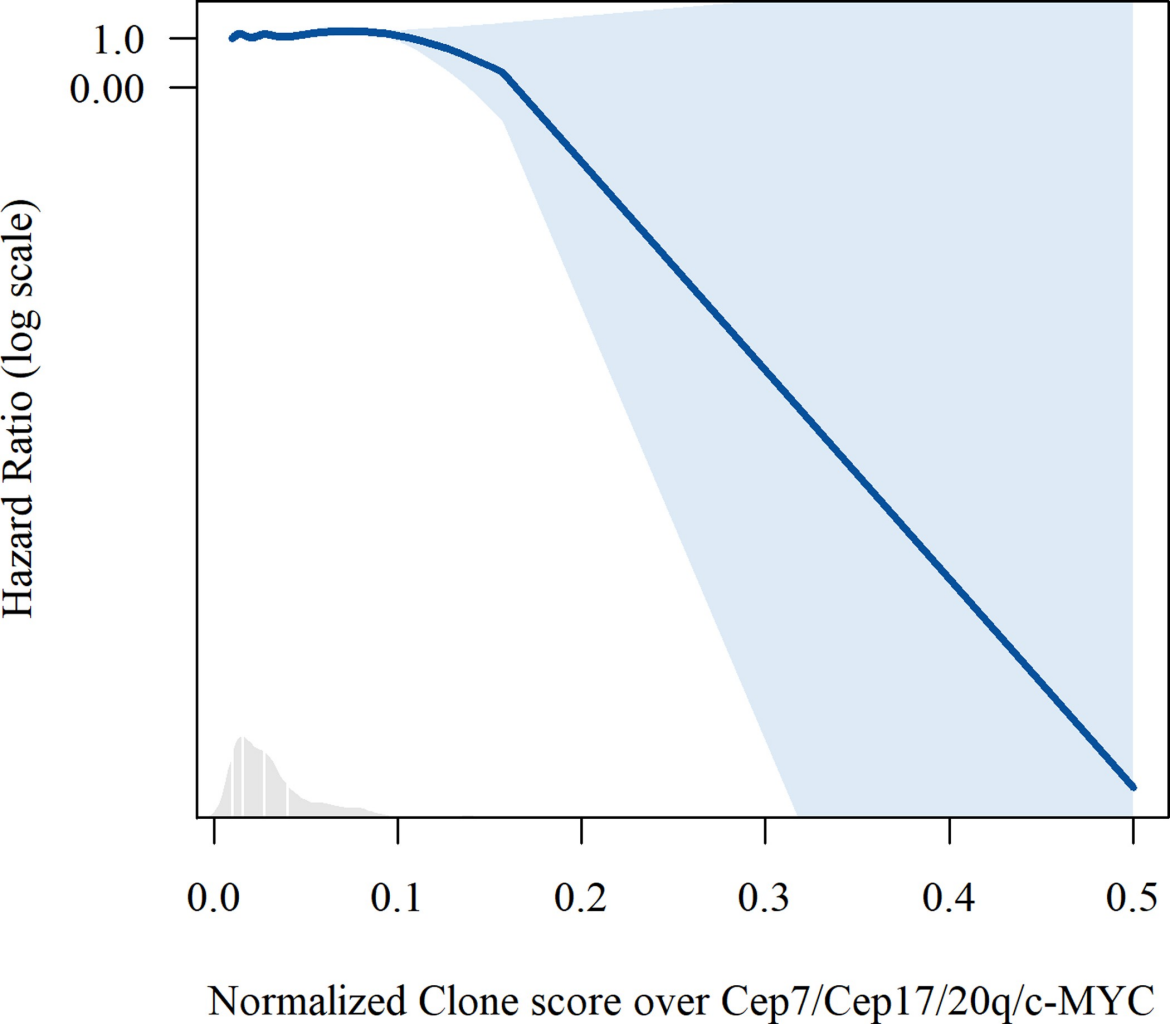
Plots of splines for normalized clone score over markers Cep7/Cep17/20q/c-MYC in the best performing multivariate model predicting progression in leave-one-out analysis.

A clinical-only model, containing age and C-Barrett length, had a median Akaike Information Criterion of 143 (IQR 121–166) and a median integrated Area Under the Curve of 0.76 (IQR 0.72–0.80). We found that the best performing model included a normalized clone score over markers CEP7, CEP17, 20q and c-MYC and the clinical covariates of age and C-Barrett length (S3 Table). Plots of splines for age and diversity score of this model are shown in Figs 2 and 3. This model had a median Akaike Information Criterion of 134 (IQR 113–157) and a median integrated Area Under the Curve of 0.88 (IQR 0.84–0.91). The p-values for all three overall tests (likelihood p<0.00, Wald p<0.00, and logrank p<0.00) were significant.

### Defining risk scores using leave-one-out analysis and Kaplan Meier analysis

**Fig 4:**
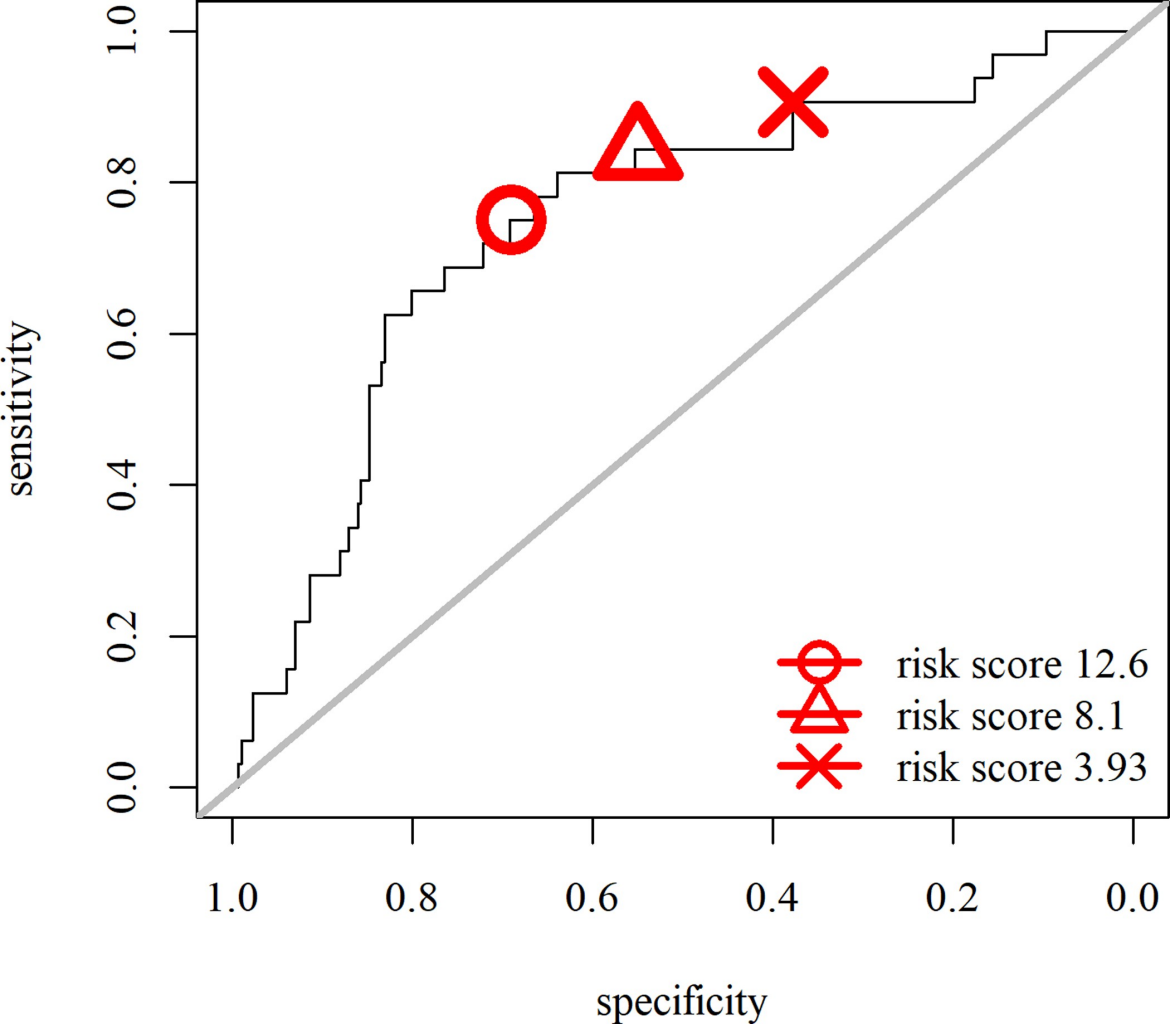
Sensitivity and specificity scores for different cut-offs for the final model with Age + C-Barrett length + normalized clone score over markers CEP7, CEP17, 20q and c-MYC. The red symbols indicate the cut-off risk scores to stratify in a high and low risk group as plotted in the Kaplan Meier curves in Figs 5–7.

Risk scores were calculated for each patient in the cohort with a leave one out validation analysis, using the variables from the best performing model (age, C-Barrett length and normalized clone score over markers CEP7, CEP17, 20q and c-MYC) as shown in Fig 1. The binary cut-offs for the risk scores for this best predicting model were chosen based on the increasing sensitivity scores as depicted by the red symbols in Fig 4.

**Fig 5:**
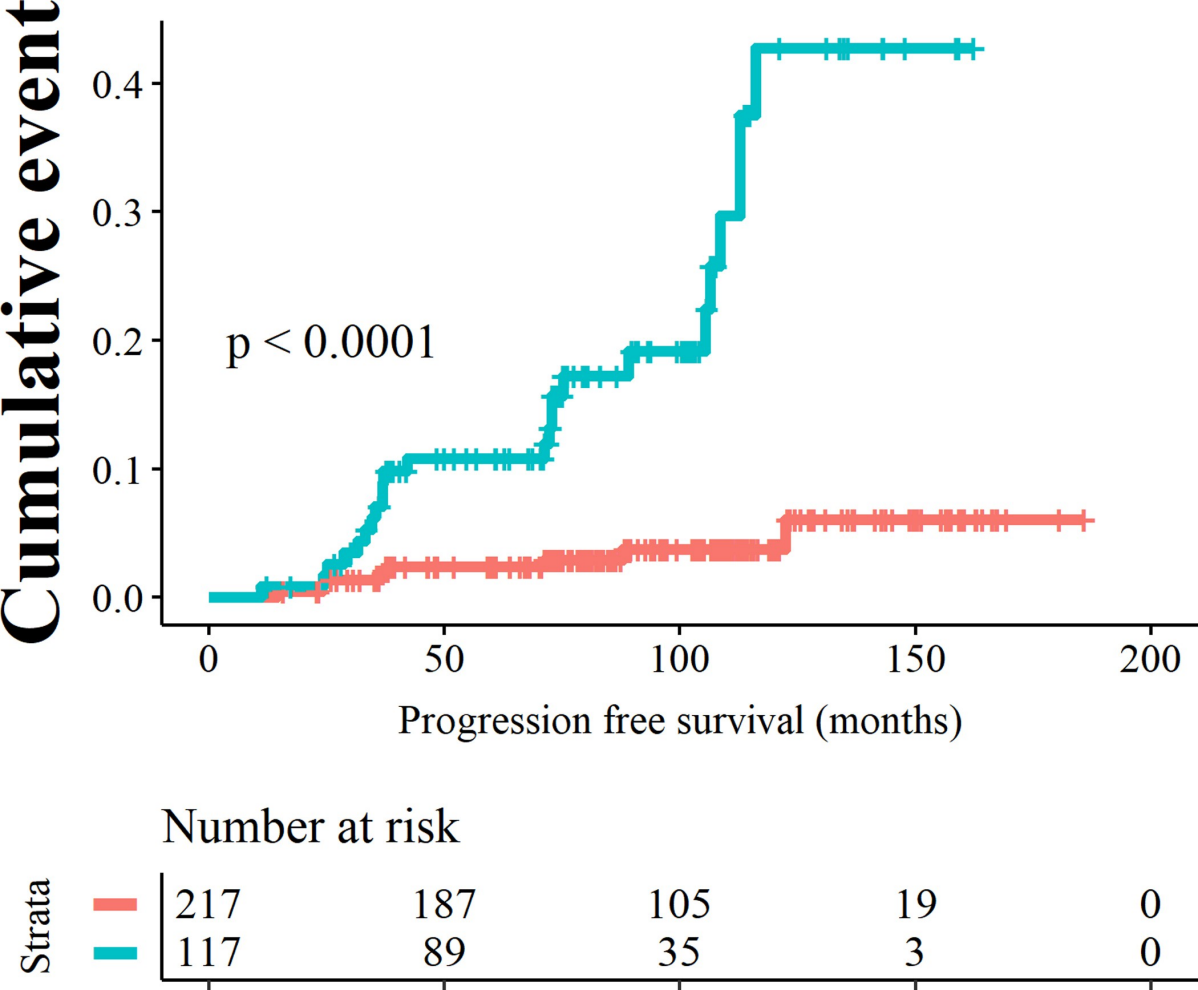
Kaplan Meier curves showing progression to HGD/EAC resulting from binary cut offs of the risk score at 12.6. Patients with a low risk score are depicted by the orange curves, patients with high risk scores are depicted by the blue curves.

Setting the risk score at 12.6 was associated with the best trade-off between sensitivity and specificity, 0.75 and 0.69 respectively (Kaplan Meier curve in Fig 5). The negative predictive value was 0.96% (95% CI 0.93 to 0. 98) and the positive predictive value was 0.21 (95% CI 0.14 to 0. 29). At this cut off, the annual progression rate was 0.5% in the low risk group versus 3.2% in the high risk group. 8 out of 217 patients versus 24 out of 117 patients progressed in the low risk and the high risk group. Patients in the high risk group have an positive and negative likelihood ratio of 2.44 (95% CI 1.87 to 3.16) and 0.36 (95% CI 0.20 to 0.66) respectively.

**Fig 6:**
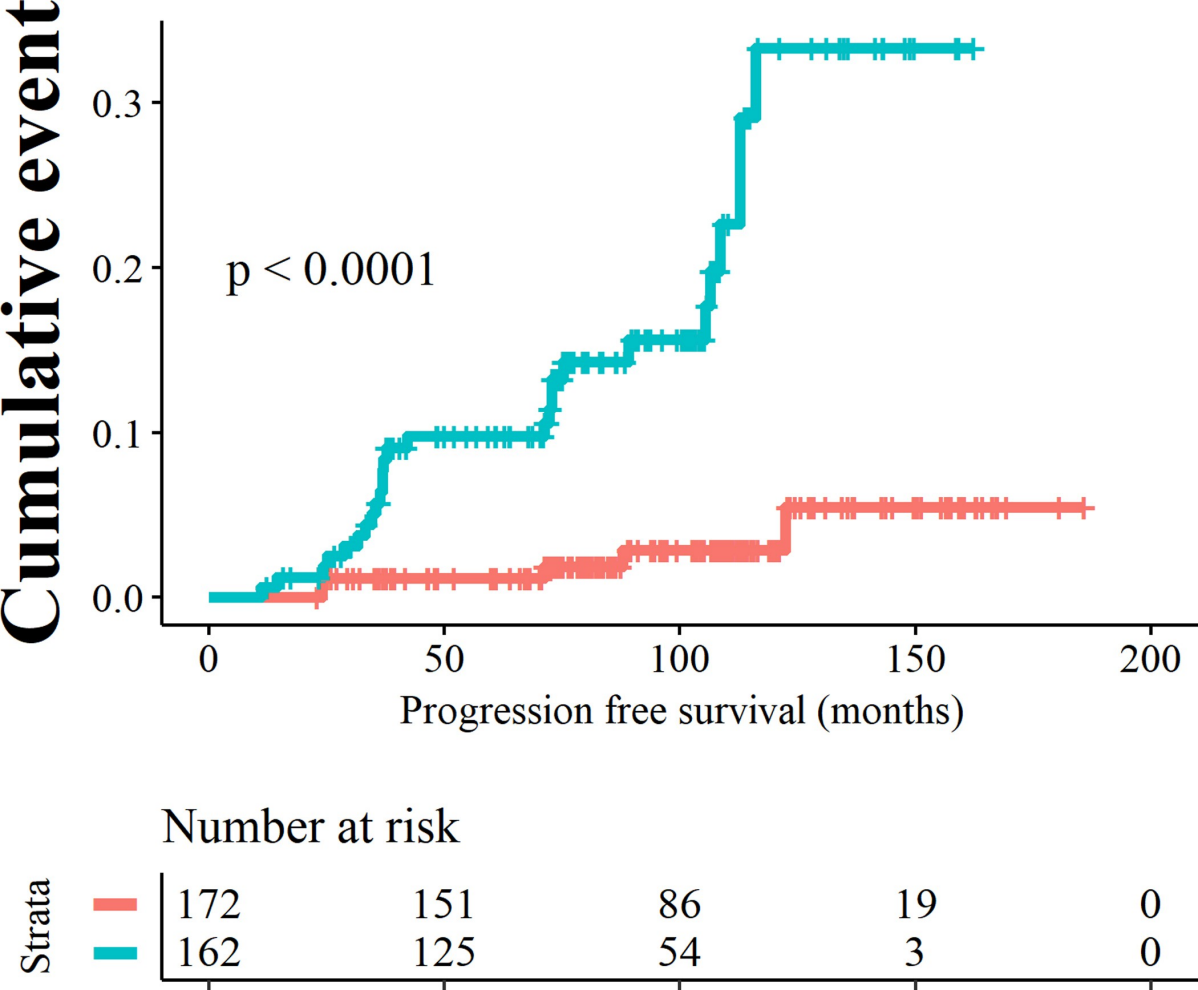
Kaplan Meier curves showing progression to HGD/EAC resulting from binary cut offs of the risk score at 8.1. Patients with a low risk score are depicted by the orange curves, patients with high risk scores are depicted by the blue curves.

Using an intermediate risk score at 8.1, the sensitivity increased to 0.84 and specificity was 0.55 (Kaplan Meier curve in Fig 6). The annual progression rate was 2.51% in high risk group versus 0.36% low risk group. In the low risk group 5 out of 172 patients progressed versus 27 out of 162 patients in the high risk group. The negative predictive value was 0.97 (95% CI 0.93 to 0.99) and the positive predictive value was 0.17 (95% CI 0.11 to 0.23). The positive and negative likelihood ratio for progression were 1.89 (95%

CI 1.55 to 2.29) and 0.28 (95% CI 0.13 to 0.64).

**Fig 7:**
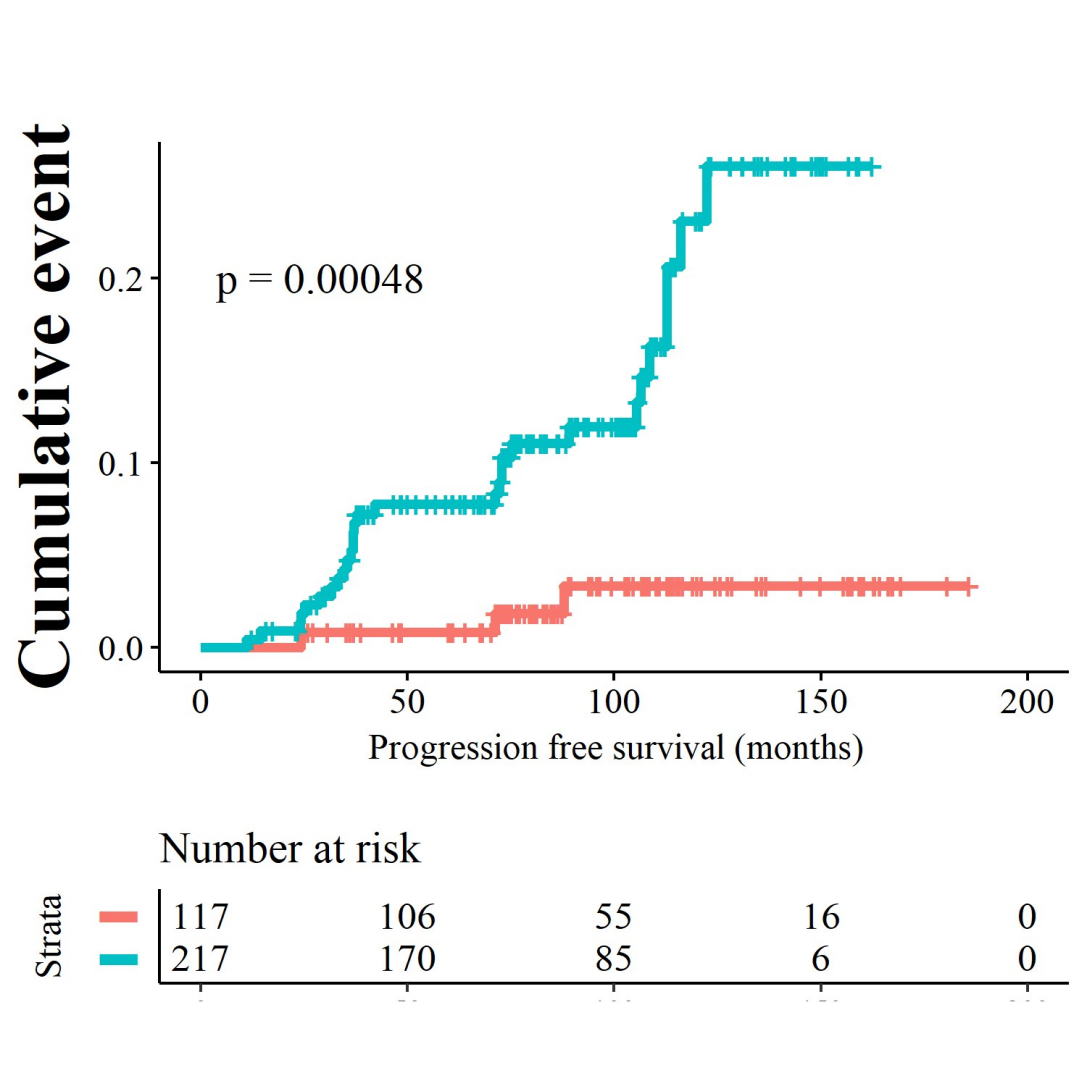
Kaplan Meier curve showing progression to HGD/EAC resulting from binary cut offs of the risk score at 3.93. Patients with a low risk score are depicted by the orange curves, patients with high risk scores are depicted by the blue curves.

However, for application of this model as a tool to adjust screening intervals in order not to miss interval cancers, a high sensitivity and negative predictive value are of higher importance. Setting the cut-off of the risk scores at 3.93, resulted in a higher sensitivity of 0.91 and specificity of 0.38 for prediction of progression. This divided the cohort in high and low risk progressors as seen in the Kaplan Meier analysis in Fig 7 (logrank test, p-value <0. 0005). The negative predictive value and positive predictive value were 0.97 (95% CI 0.93 to 0.99) and 0.13 (95% CI 0.09 to 0.19) respectively. In this model, NDBE patients with a high risk score, have a positive and negative likelihood ratio of 1.46 (95% CI 1.26 to 1.68) and 0.25 (95% CI 0.08 to 0.74). In the low risk group, 3 out of 117 patients progressed, in the high-risk group 29 out of 217 progressed. The annual progression risk to HGD/EAC in the low risk group was 0.31% versus 1.92% in the high risk group.

## Discussion

One of the aims of this study was to investigate the accuracy of genomic and clinical predictive biomarkers in a Dutch cohort of NDBE patients, as there is an unmet need for such biomarkers to increase the efficacy of surveillance programs. To decrease costs, endoscopic screening of the majority of NDBE patients have been prolonged to 5-yearly intervals, increasing the risk for interval advanced stage cancers. The aim of this study was to specifically address this problem by determining if biomarkers are able to predict progression over longer time intervals and as such can be used to improve patient stratification. These biomarkers include individual marker scores and clonacity measurements.

In this cohort of NDBE patients with a median follow-up time of 86.5 months (IQR 39.7), we found that the model, which included the diversity measure defined as the normalized clone score over the FISH markers CEP7, CEP17, 20q and c-MYC and the clinical variables of age and C-Barrett length, was superior to other models with regard to predicting progression to HGD/EAC. The purpose of this model was to be able to improve the surveillance algorithm of NDBE patients. This requires to more safely extend surveillance intervals for those with the lowest risk to progress to cancer and to diminish the risk on advanced stage interval cancers of those who are at higher risk to develop cancer. When choosing a relatively low risk score of 3.93 as a cut off to select for high and low risk patients, the model yields a sensitivity of more than 90% and a negative predictive value of almost 98%. This is highly beneficial for the safety of the at-risk patients, and of patients that would test negative for the test. The down side is that the low specificity (around 40%) leads to many cases which will falsely test positively. Despite the low risk for cancer, these patients will undergo more frequent surveillance, which is associated with higher costs. Therefore, the decision on the exact ‘test positive value’ of the model will greatly depend on the amount that one is willing to spend per life year gained. Such a cost effectiveness study would be the next step before the clinical implementation of the model.

The prediction performance in univariate analyses of the variables reported here, are in line with earlier studies on this cohort, in which the same diversity variable, namely the normalized clone score over markers CEP7, CEP17, 20q (20q13.2) and c-MYC (8q24.12) was the most significant predictor of progression [7]. Similarly, increased clonal abundancy was found to be associated with an increased risk of progression.

Earlier, we hypothesized that it is essential to carefully choose the particular markers that should be used for diversity calculations, as some genetic aberrations are of more importance for malignant progression than others, and also to increase cost-effectiveness when implementing these biomarkers in clinical practice. Previously, we found that diversity measurements on clones defined by c-MYC and clones defined by cep7 were more prognostic than others [7]. In the current analysis, we also found that normalized clone score calculated over clones defined by the 3 markers TP53/ERBB2/Cep17 was a good predictor in univariate analysis. However, in multivariate analyses, the model with age, C-Barrett length and the NC over markers CEP7, CEP17, 20q and c-MYC proved to be superior, while a model including C-Barrett length, age and the NC score of 3 markers TP53/ERBB2/Cep17 was the second-best performing model based on the median integrated Area Under the Curve. The median integrated area under the curve increased from 0.76 to 0. 88 when comparing a clinical-only model to the superior multivariate model.

In the current and previous analyses of this cohort and studies on other BE cohorts, age and C-Barrett length were found to be (borderline) significant predictors of progression in univariate analyses [7, 8] [19–21]. The individual marker scores, that previously showed good prediction performance in a multivariate model containing age, C-Barrett length and markers CDKN2A, c-MYC and aneusomy with an area under the curve of 0.76 (95% CI 0.66 to 0.86), were inferior to diversity measurements added to clinical characteristics in multivariate modeling in the current study after extended follow up.

The strength of our study is that our analyses are performed on a cohort of patients that was prospectively followed over an extended period of time. The median follow period was 86.5 months with an interquartile range of 39.7. The consequence of our prospective approach is that we had a relatively low number of progressors. This was to be expected given the low progression rate of NDBE patients. The downside is that the relatively low number of progressors (32) restricted the multivariate model to include no more than 3 variables in order to prevent overfitting of the model. A larger cohort with more progressors would be needed to investigate models with more than three markers. For example a model with all (borderline) significant variables in univariate analyses including age, C-Barrett length, Cep7 gain and clonicity scores from the both marker sets would require at least 50 events (progressors) and thus a much larger cohort of NDBE patients.

This study is also unique, as the patients participating in this prospective study represented a NDBE population at baseline, resembling 95% of the Barrett’s surveillance population. Besides our study, there are no large prospective biomarker studies focusing solely on these NDBE patients. After our previous studies, most of the patients in this cohort were still under active endoscopic surveillance. We report an annual progression risk of 1.3% to HGD/EAC and 0.85% to EAC, which seems to be higher as compared to the general literature. Since a large amount of our patients were included in a tertiary referral center, our cohort is biased towards patients with longer Barrett segments (C-Barrett length median 2.0 cm, IQR 4.0 M-Barrett length median 4.0 cm, IQR 4.0).

Progression rates of longer BE segments to EAC are notably higher and have found to be between 0.62% to 0.93%, which is in line with our data [22–25].

For now, the studied genomic characteristics seem suitable predictive biomarkers for progression risk in NDBE patients, as they remain invariant over time [7]. Based on the results from this study, we propose to stratify patients with NDBE into a low risk and a high risk for progression. This would allow to perform a cost-benefit analysis and to more exactly decide which risk score to use for more efficient stratification of patients in different surveillance arms. Current surveillance regimens which are relatively intense (once per 3 years) should be maintained in the patients with a high risk for progression, to prevent an increase of advanced stage interval cancers due to relaxation of surveillance intervals of the entire group of BE. Patients with a low risk of progression will benefit from undergoing less burdensome surveillance endoscopies. Finally, implementation of these markers in clinical practice will enable increasing surveillance intervals without consequent increase of advanced stage EAC incidence associated with a dismal prognosis, thereby optimizing patient management and improving cost-effectiveness of our current surveillance strategies [26].

## Supporting information

S1 Table Outcomes of the best performing clonal diversity scores for 1, 2, 3 and 4 markers from set 1^†^. (PDF)

S2 Table Outcomes of the best performing clonal diversity scores for 1, 2, 3 and 4 markers from set 2^†^. (PDF)

S3 Table Multivariate prediction model including age, C-Barrett length and Normalized Clones over a set of markers according to a leave-one-out analysis. (PDF)

S4 Table Data set underlying results in this manuscript. (XLSX)
