# Supplementary material for: A genomic biomarker-based model for cancer risk stratification of non-dysplastic Barrett’s esophagus patients after extended follow up; results from Dutch surveillance cohorts
Source: PLoS One. 2020 Apr 13;15(4):e0231419. doi: 10.1371/journal.pone.0231419 (PMC7153893; doi:10.1371/journal.pone.0231419)
Supplement: S1 Table — (PDF) [file pone.0231419.s001.pdf]

Model	Median iAUC^ 1000 rep	Median AIC^ 1000 rep	P value wald*	P value likelihood ratio*	HR*	95% CI*
1 marker:<br>Shannon CDKN2A	0.55	148	0.3	0.3	0.28	0.03-2.76
2 markers:<br>Simpson C CDKN2A/ ERBB2	0.55	148	0.4	0.4	0.25	0.01-7.68
**3 markers:<br>NC TP53/ERBB2/CEP17**	**0.56**	**147**	**0.03**	**0.07**	**821491**	**3.44-1.96e+11**
4 markers:<br>NC<br>CDKN2A/TP53/ERBB2/CEP17	0.53	149	0.2	0.3	883.3	0.03-28046568

†markers CDKN2A/TP53/ERBB2/ CEP17

^ Obtained with bootstrapping

* Results from univariate Cox proportional hazards models. Bold values indicate statistical significance (P<0.05).

iAUC, integrated Area Under the Curve; AIC, Akaike's Information Criterion; HR, Hazard Ratio; CI, Confidence Interval; Shannon, Shannon diversity score; Simpson C, Simpson Compliment diversity score; NC, Normalized Clone score
