# Supplementary material for: A genomic biomarker-based model for cancer risk stratification of non-dysplastic Barrett’s esophagus patients after extended follow up; results from Dutch surveillance cohorts
Source: PLoS One. 2020 Apr 13;15(4):e0231419. doi: 10.1371/journal.pone.0231419 (PMC7153893; doi:10.1371/journal.pone.0231419)
Supplement: S2 Table — (PDF) [file pone.0231419.s002.pdf]

Model	Median iAUC^ 1000 rep	Median AIC^ 1000 rep	P value wald*	P value likelihood ratio*	HR*	95% CI*
1 marker:<br>NC CEP7	0.59	152	0.7	0.7	6.93	0.00-65306
2 markers:<br>NC CEP7/20q	0.63	151	0.4	0.5	19.87	0.01-26655
3 markers:<br>NC CEP7/20q/c-MYC	0.62	149	0.2	0.3	57.59	0.20-16823
4 markers:<br>NC CEP7/CEP17/<br>20q/c-MYC	0.61	150	0.1	0.2	66.79	0.30-15024

†markers CEP7/CEP17/20q/c-MYC

^ Obtained with bootstrapping

* Results from univariate Cox proportional hazards models.

iAUC, integrated Area Under the Curve; AIC, Akaike's Information Criterion; HR, Hazard Ratio; CI, Confidence Interval; NC, Normalized Clone score
