# Supplementary material for: A genomic biomarker-based model for cancer risk stratification of non-dysplastic Barrett’s esophagus patients after extended follow up; results from Dutch surveillance cohorts
Source: PLoS One. 2020 Apr 13;15(4):e0231419. doi: 10.1371/journal.pone.0231419 (PMC7153893; doi:10.1371/journal.pone.0231419)
Supplement: S3 Table — (PDF) [file pone.0231419.s003.pdf]

Variable	Variable type	Mean	P value*	HR*	95% CI*
Age (df=4)1	0-25% (19- 51.25 years)		0.18	1.75e+12	2.77e-06 - 1.10e+30
Age (df=4)2	25-50% (51.25- 60 years)		0.13	2.36e+08	3.19e-03 - 1.74e+19
Age (df=4)3	50-75% (60- 67 years)		0.18	4.77e+24	9.87e-12 - 2.30e+60
Age (df=4)4	75-100% (67- 87 years)		0.28	2.12e+04	3.44e-04 - 1.30e+12
C-Barrett length	continuous	2.72	0.03	1.12	1.01 - 1.25
Normalized clones CEP7/CEP17/20q/c-MYC (df=6) 1	0-16.7% (0.01 - 0.013)		0.46	0.32	1.47e-02 - 6.86
Normalized clones CEP7/CEP17/20q/c-MYC (df=6) 2	16.7-33% (0.013-0.020)		0.04	20	1.24 - 322
Normalized clones Cep7/Cep17/20q/c-MYC (df=6) 3	33-50% (0.020- 0.027)		0.94	0.89	4.74e-02 - 16.7
Normalized clones CEP7/CEP17/20q/c-MYC (df=6) 4	50-66.7% (0.027-0.033)		0.01	1.19e+12	1.62e+03 - 8.77e+20
Normalized clones CEP7/CEP17/20q/c-MYC (df=6) 5	66.7-83.3% (0.033-0.050)		0.09	1.83e-70	3.74e-151 - 8.95e+10
Normalized clones Cep7/Cep17/20q/c-MYC (df=6) 6	83.3-100% (0.050-0.5)		0.07	3.79e-153	9.45e-320 - 1.52e+14

* Results from multivariate Cox proportional hazards model.

HR, Hazard Ratio; CI, Confidence Interval; df, degrees of freedom

Splines were used to divide the variables age and diversity in multiple segments (segment boundaries are described in column “variable type”).
